# Charge against a Medical Practitioner

**Published:** 1851-08

**Authors:** 


*Charge against a Medical Practitioner.*—A curious case has recently been tried at Gloucester, involving the reputation of a medical practitioner, Mr. Sheldon, of Cheltenham. It consisted of a charge of making improper examinations of a young girl, aged seventeen. At first the matter was heard before the magistrates of Cheltenham where it was dismissed, the Bench stating it as their opinion that Mr. Sheldon left the court "without the slightest stain upon his character." The recent trial for an assault came on before a special jury of the most respectable chaiacter.

The only witness was the prosecutrix herself, who thus details one of the interviews with Mr. Sheldo ., and which constituted part of the alleged assault:—

"He examined me on that occasion, and told me to come again on the following Tuesday, and I went again on the 26th. He told me to take my clothes off; I did take all off, but my under things, all but my chemise and my flannel; he then said, 'let that down.' I did let it down. Mr. Sheldon felt my bowels and my side. I did not let it further down than the middle of my stomach, and then he felt my bowels and my side. He felt my pulse, and said it was nineteen to the dozen. He said, how frightened you are! He asked me a great many questions about my family. I went on the following Tuesday, about ten o'clock; the woman servant let me in. I went into the surgery; an elderly person was there. Mr. Sheldon said to the woman 'I must trouble you to go out.' She went out, and I remained there. Mr. Sheldon drew the blind down, and he then told me to take my things off. I said, 'What! again, sir?' He said, 'yes, again, and again; take it off, and dont mind me at all; don't be frightened at me, for I cannot tell you if you get better without.' I took off my dress, and said, 'Not take off my stays, sir, and other things?' He said, 'Yes, you must take the whole of it off, and don't mind me, any more than if yourself was in the room.' I did take off all but my under things. Mr. Sheldon said, 'Let that down; (that was my flannel and under garment.) He had his hands on them. I said, 'I cannot let that down again sir.' He said, 'Well, I cannot tell without.' He pushed it down at the same time with great force. I held it, but Mr. Sheldon pushed it down. I pulled it up again, but he again pushed it down; it was tied, but Mr. Sheldon's force slipped the string. I think it broke; yes, it did break, as he pushed it down. I did not wish him to push it down. When that was pushed down, I did not let it go out of my hand, but Mr. Sheldon held it down below my entire body, down to about my thighs, so that my person was exposed. I tried to pull it up. Mr. Sheldon felt my bowels and my side, and tried to take further liberties with his right hand."

Other acts of familiarity were said to have followed, but it was most remarkable that the girl described two or three other interviews with Mr. Sheldon in almost the same words as the above. Scarcely a word of alteration occurred in her description of the several meetings. It appeared that the girl consulted Mr. Sheldon as a dispensary patient, for a debilitated constitution, and pain in the side; and she had ulcers on both ancles, and other repulsive symptoms; but she averred that not only did these assaults take place, but that Mr. Sheldon was so smitten with her as to propose private meetings in the neighborhood of Cheltenham, and even elopement to Bristol or Clifton, or a permanent connexion; Mr. Sheldon being a respectable married man, having a wife and family, and in an extensive practice.

A large number of professional and other witnesses were called, including the rector of Cheltenham, (the Rev. F. Close,) all of whom bore the most explicit testimony to the honorable and upright character of Mr. Sheldon through a long series of years. While this evidence was proceeding, the jury expressed some equivocal signs of their desire to bring the case to a conclusion.

The following was the termination of the trial, than which we have seldom seen anything more satisfactory :—

" Dr. Collings Robinson was called, but not answering immediately to his name,—

Mr. Keating said, Really, my lord, I won't trouble the jury with any further witnesses.

Mr. Justice Patteson: It is quite unnecessary. Gentlemen of the Jury, I don't know what *you* think of this case.

The Foreman of the Jury : My Lord, we were just about to say that we would not trouble your lordship to sum up, as we have already agreed to give a verdict for the defendant, if that would be a proper course. I have further to say that we consider that his character is altogether unstained. (Cheering.)

His Lordship : I entirely agree with you, gentlemen, and I don't believe there is one word of truth in the charge from begining to end. (Applause.) Mr. Sheldon will go out of this court without the slightest stain upon his character. No man would be safe, gentlemen, if these sort of stories were to be believed. You cannot place any reliance upon a witness who swears to conversations which took place time after time in precisely the same words. It is monstrous.

As defendant was leaving the court, the jurymen leaned over the side of their box, and shook him cordially by the hand.

Medical men must of course bear their share of slander and misrepresentation, but it does seem monstrous that on the unsupported evidence of a girl who, according to her own statement, goes again and again to be exposed and assaulted in a disgraceful manner, a respectable medical man should be put on his formal trial for such alleged offences. We cannot understand how a grand jury could have returned a true bill in such an absurd case.

However, the matter has been tried. Every medical man in the kingdom should congratulate Mr. Sheldon on the result, though in his case it could not have been otherwise than it was. There is not a practitioner to be found against whom similar accusations might not be foisted to-morrow, if persons wicked and designing enough to make such charges could be found. As is generally the case with falsehood, too much was attempted to be proved, and the clear-sighted judge at once saw through the trickery and improbability, not to say impossibility, of the charges advanced.—*London Lancet.*

